# Gadofullerene nanoparticles for robust treatment of aplastic anemia induced by chemotherapy drugs

**DOI:** 10.7150/thno.46794

**Published:** 2020-05-23

**Authors:** Wang Jia, Mingming Zhen, Lei Li, Chen Zhou, Zihao Sun, Shuai Liu, Zhongpu Zhao, Jie Li, Chunru Wang, Chunli Bai

**Affiliations:** 1 Beijing National Laboratory for Molecular Sciences, Key Laboratory of Molecular Nanostructure and Nanotechnology, CAS Research/Education Center for Excellence in Molecular Sciences, Institute of Chemistry, Chinese Academy of Sciences, Beijing 100190, China.; 2 University of Chinese Academy of Sciences, Beijing 100049, China.

**Keywords:** gadofullerene nanoparticles, aplastic anemia, reactive oxygen species, erythropoiesis, erythrocyte maturation

## Abstract

Aplastic anemia (AA) is characterized as hypoplasia of bone marrow hematopoietic cells and hematopenia of peripheral blood cells. Though the supplement of exogenous erythropoietin (EPO) has been clinically approved for AA treatment, the side-effects hinder its further application. Here a robust treatment for AA induced by chemotherapy drugs is explored using gadofullerene nanoparticles (GFNPs).

**Methods**: The gadofullerene were modified with hydrogen peroxide under alkaline conditions to become the water-soluble nanoparticles (GFNPs). The physicochemical properties, *in vitro* chemical construction, stability, hydroxyl radical scavenging ability, *in vitro* cytotoxicity, antioxidant activity, *in vivo* treatment efficacy, therapeutic mechanism and biological distribution, metabolism, toxicity of GFNPs were examined.

**Results**: GFNPs with great stability and high-efficiency antioxidant activity could observably increase the number of red blood cells (RBC) in the peripheral blood of AA mice and relieve the abnormal pathological state of bone marrow. The erythropoiesis mainly includes hemopoietic stem cells (HSCs) differentiation, erythrocyte development in bone marrow and erythrocyte maturation in peripheral blood. The positive control-EPO promotes erythropoiesis by regulating HSCs differentiation and erythrocyte development in bone marrow. Different from the anti-AA mechanism of EPO, GFNPs have little impact on both the differentiation of HSCs and the myeloid erythrocyte development, but notably improve the erythrocyte maturation. Besides, GFNPs can notably decrease the excessive reactive oxygen species (ROS) and inhibit apoptosis of hemocytes in blood. In addition, GFNPs are mostly excreted from the living body and cause no serious toxicity.

**Conclusion**: Our work provides an insight into the advanced nanoparticles to powerfully treat AA through ameliorating the erythrocyte maturation during erythropoiesis.

## Introduction

Aplastic anemia (AA), as one kind of acquired myeloid hematological system diseases, is characterized by the dysfunction of the hypocellular marrow and pancytopenia 1-3. Meanwhile, the hemoglobin (HGB) lowering than 110 g/L in peripheral blood usually acts as an intuitive criterion of AA. The decreasing level of HGB is closely connected with the descending counts of red blood cells (RBC), which is commonly induced by the damaged function of erythropoiesis. Erythropoiesis is a complex process, including the differentiation of hematopoietic stem cells (HSCs), the development of erythrocyte and the maturation of erythrocyte 4,5, and the first two stages occur in bone marrow. The multifunctional HSCs as a sole source of hemocytes could be self-renewal and differentiate into various blood cells. They develop from nucleated red blood cells (NRBC) into reticulocytes (Retic) in bone marrow, which is called the erythrocyte development 6,7. The Retic further matures in peripheral blood, developed into mature RBC.

Although the pathogenesis of AA is poorly understood, it closely related to chemical drugs, radiation, viral infection, genetic factors and so forth 8-10. Chemotherapy is the most common cause of AA 1,2,8. It causes direct injury towards HSCs and the hematopoietic microenvironment by chemotherapy drugs, resulting in AA. Currently, exogenous supplement with erythropoietin (EPO, produced by kidney) could stimulate erythropoiesis in bone marrow 5,11,12. But this potentially causes tissue hypoxia, venous thrombosis, liver, and heart failure by EPO treatment. Additionally, uses of hematopoietic stem cell transplantation (HSCT) and immunosuppressive therapy (IST) for AA treatment also have numerous side-effects, e.g. low efficiency, relapses, unfavorable prognosis, high cost and others 9,10,13,14. Thus, there is still lack of an effective and low toxicity therapeutic strategy for AA.

To date, nanomaterials mediated anti-oxidative therapy is emerging as a novel strategy in disease treatment through scavenging excessive reactive oxygen species (ROS) 15-19. Among them, fullerenes and gadofullerene possess excellent capacities for scavenging ROS, and have exhibited superior effects on the treatments of many diseases associated with ROS, e.g. tumor, neurodegenerative disease, hepatic injury, pulmonary fibrosis, and so forth 20-25. In addition, our previous studies showed that gadofullerene nanoparticles (GFNPs) could be specially accumulated into mice bone marrow by intravenous injection, promoting the content of white blood cells (WBC) and protecting against myeloid oxidative injury during chemotherapy in mice 22.

In this work, we further demonstrated that GFNPs have a potent anti-AA effect in mice by boosting the level of RBC and HGB. Mechanically we investigated the effects of GFNPs on the three stages of erythropoiesis. Our results revealed that GFNPs could not significantly regulate the differentiation of HSCs and the myeloid erythrocyte development, but notably promote the erythrocyte maturation, lower the level of excessive ROS and inhibit apoptosis of blood cells in peripheral blood of AA mice. Furthermore, the GFNPs were mostly metabolized out of the body without causing obvious damage. This work provides a promising strategy to develop advanced nanomedicine for robust AA treatment.

## Results and Discussion

### Synthesis and Characterizations of GFNPs

**Figure 1:**
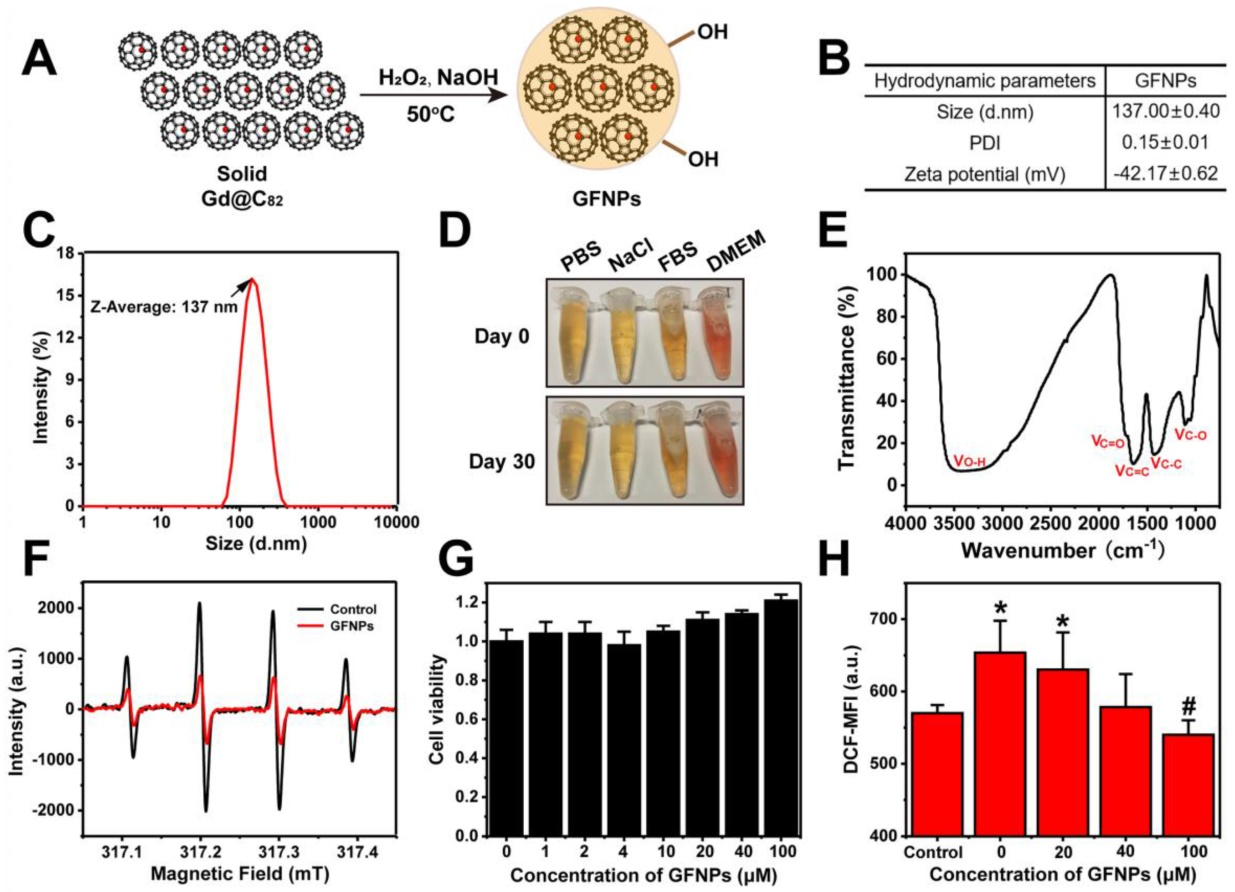
** Preparation and Characterization of GFNPs. (A)** Schematic diagrams of the synthesis of GFNPs. **(B)** The average size, polydispersity index (PDI) and Zeta potential of GFNPs in water. **(C)** Hydrodynamic size distribution of GFNPs by DLS. **(D)** Optical photographs of GFNPs in PBS (1x), 0.9 % NaCl, FBS and DMEM-H, on day 0 (up) and day 30 (down) after synthesized. **(E)** The FT-IR spectrum of GFNPs. **(F)** The ESR spectra of •OH captured by DMPO after treated with the ultrapure water (black line) or 30 µM GFNPs (red line). **(G)** The FDC-P1 cell viability after treatment with GFNPs (0, 1, 2, 4, 10, 20, 40, 100 µM). **(H)** The intracellular ROS levels of FDC-P1 cells treated with different concentrations of GFNPs (0, 20, 40, 100 µM) after treatment with H_2_O_2_ (400 µM) in dark for 1 h. (n≥3;^ *^p < 0.05, vs Control group; ^#^ p < 0.05, vs only H_2_O_2_-treatment group).

In this study, we adopted a simple solid-liquid reaction to modify gadofullerene to become water-soluble according to our previous works 26,27. Briefly, gadofullerenes reacted with 30% hydrogen peroxide under alkaline conditions at 50 ^o^C for about 30 min (Figure 1A). The hydroxyl bonding on the carbon cage made Gd@C_82_ molecules have a good dispersion in aqueous solution. Atomic force microscope (AFM) was to investigate the morphology of GFNPs, and Figure S1 showed that GFNPs had a diameter of ca. 50-80 nm and height of ca. 6-12 nm. Meanwhile, dynamic light scattering (DLS) was for the characterization of hydrodynamic parameters. Results in Figure 1B showed that the hydrodynamic size of GFNPs was 137.0±0.4 nm, owing to the strong interaction between hydrogen bonding of GFNPs and water molecules of hydroxyl groups. Besides, the hydrodynamic size distribution (Figure 1C) and the small polydispersity index (PDI, 0.15±0.01, Figure 1B) of the GFNPs indicated that GFNPs have a uniform size distribution. The previous studies have showed that the stability of nanoparticle is mainly influenced by its surface charge, and the Zeta potential of which between -30 mV~30 mV tends to form aggregates in aqueous solution 28. In present study, we demonstrated that the Zeta potential of GFNPs is -42.17±0.62 mV (Figure 1B), the absolute value of which is greater than 30 mV, thus we suggested that GFNPs nanoparticle was relatively stable. Additionally, we have verified the high stability of GFNPs in PBS, 0.9% NaCl, FBS and dulbecco's modified eagle medium (DMEM) by observation the optical photographs on day 0 and day 30 after the synthesis, and there was no noticeable precipitate after 30 days (Figure 1D).

Fourier transform infrared spectrum (FT-IR) provided the information of the chemical structure of GFNPs (Figure 1E), and three main peaks situated at 1350-1400, 1600, and 3400 cm^-1^ refers to the stretching bands of C-C or C-O and vibrational bands of C=C, O-H, respectively. Meanwhile, the weak C=O peak located at 1750-1700 cm^-1^ was possibly corresponding to the pinacol rearrangement and subsequent keto-enol isomerization. X-ray photoelectron spectroscopy (XPS) was for the average composition of GFNPs, and the data (Figure S2) showed that the C1s peaks of GFNPs included 1 C-C (or C=C), 2 C-O, and 3 C=O, which were 45%, 32%, and 23%, respectively. Together, the average composition of GFNPs was calculated as Gd@C_82_(O)_~19_(OH)_~26_.

Considering hydroxyl radical (•OH) is one of the most common types of ROS connected with AA 29-31, we adopted the electron spin-resonance (ESR) spectrum to test the hydroxyl radical-scavenging capacity of GFNPs. H_2_O_2_ (20 μL,100 mM) was for generating •OH after UV-irradiation for 4 min, and the spin trap 5-dimethyl-1-pyrroline-N-oxide (DMPO, 40 μL,100 mM) acted as a trapping agent for •OH. DMPO-OH adduct performed as a typical four-line ESR signal (1:2:2:1 quartet) in Figure 1F. Data showed that only 30 μM GFNPs could scavenge almost 65% hydroxyl radicals, which illustrated that GFNPs have a superior anti-oxidative capability.

Further, we incubated FDC-P1 cells (normal mice bone marrow cells) with GFNPs to testify the cytotoxicity and antioxidant capacity of GFNPs. Firstly, we found that the cell viability of FDC-P1 cells was undiminished but a little increased after incubated with GFNPs within 100 μM for 24 h (Figure 1G), indicating that GFNPs have no obvious cytotoxicity within at least 100 μM. Subsequently, we employed the method of flow cytometry (FCM) to investigate the antioxidant capacity of GFNPs in the cellular level. H_2_O_2_ was used for generating ROS in FDC-P1 cells and DCFH-DA (a ROS sensitive probe) was to evaluate the intensity of ROS. After incubated with GFNPs (20, 40, 100 μM) for 3 h, there was an obvious reduction of the ROS level which was produced by H_2_O_2._ Thus, we drew a conclusion that GFNPs evidently lowered the excessive ROS levels of FDC-P1 cells induced by H_2_O_2_ (Figure 1H).

Besides, we conducted the biosafety studies* in vivo*, which is a vital factor for future use in clinical. Figure S3 showed that the body weights and some of major hematological indices (such as RBC, HGB, and hematocrit (HCT)) of mice had no noticeable changes in the GFNPs treated groups compared with the controls. Moreover, the main organs including heart, liver, spleen, lung and kidney had no obvious pathological change on day 15 after treated with GFNPs for six times. Together, all these data indicated that GFNPs had superior capability of not merely the high efficiency anti-oxidation, but also the preeminent biocompatibility.

### The effects of GFNPs on AA induced by chemotherapy drugs in mice

**Figure 2:**
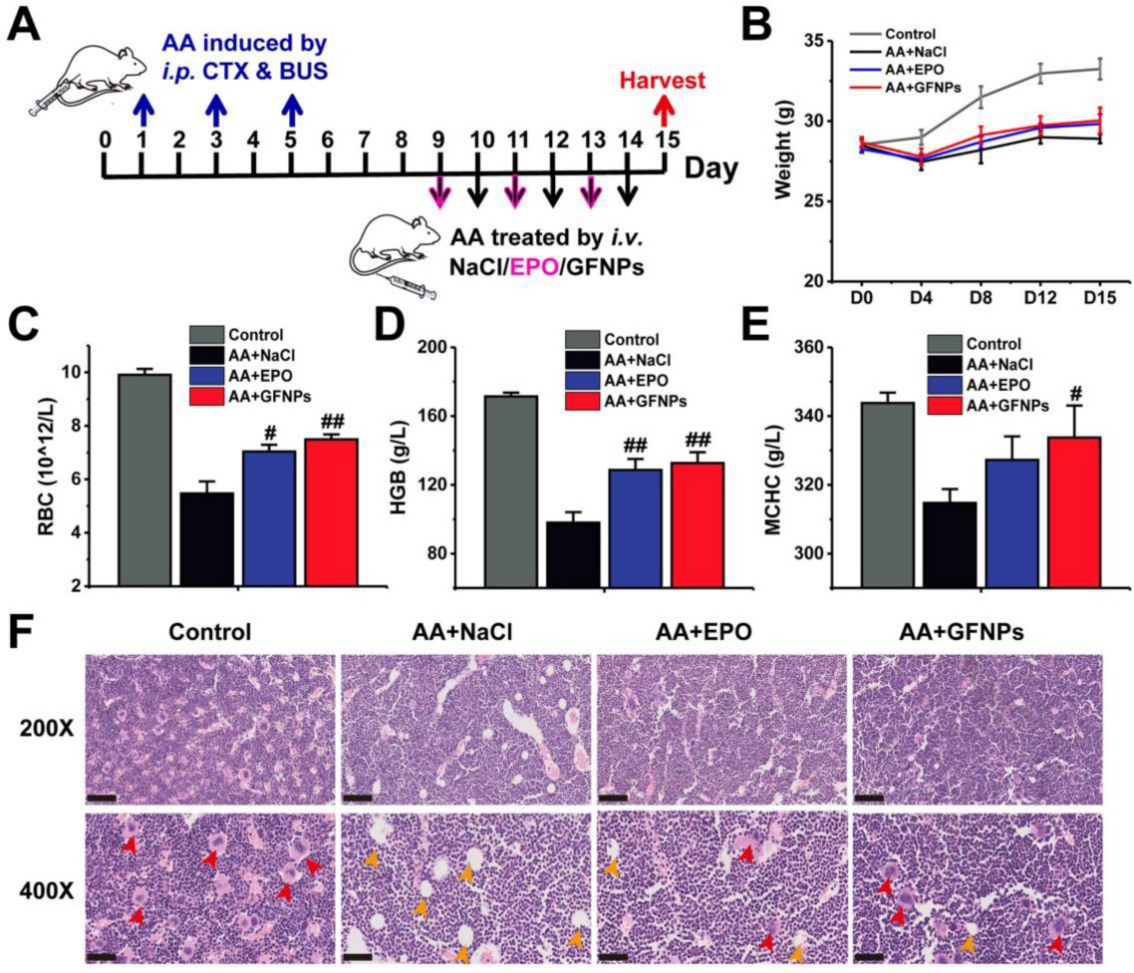
** The anti-AA effects of GFNPs *in vivo*. (A)** Schematic diagram depicted the experimental protocol of anti-AA. **(B)** The body weights during 15 days observation. **(C-E)** The levels of red blood cells (RBC), hemoglobin (HGB) and mean corpuscular hemoglobin concentration (MCHC) at the end of treatment. **(F)** Light microscopy of H&E sections of mice bone marrow at 200X and 400X magnification. The red arrow indicated hematopoietic cells of red pulp, and the yellow arrow indicated adipose in yellow pulp. Scale bar = 100 µm (Up; 200X) and 50 µm (Bottom; 400X). (n=5; ^#^ p < 0.05, ^##^ p < 0.01, vs AA+NaCl group).

To evaluate the anti-AA effects of GFNPs, we established an AA mice model by three times of intraperitoneal (i.p.) administration with CTX & BUS (Figure 2A). The HGB in AA mice was observably decreased below 110 g/L by hematological examination, indicating we successfully obtained the AA mice models. Then the AA mice were further intravenously (i.v.) treated with saline, EPO (6×10^4^ IU/kg/d, 3 days) and GFNPs (60 mM/kg/d, 6 days), respectively (n = 5). The weights of AA mice were sharply decreased, and conversely, the AA mice treated with GFNPs and EPO showed quite increases in body weight after one-week observations (Figure 2B). To quantitatively evaluate the anti-AA effects of GFNPs, we took systematic hematological examinations at the end of the experiment. As shown in Figure 2C-E, there were significant decreases of the RBC, HGB and mean corpuscular hemoglobin concentration (MCHC) in AA mice. Interestingly, the levels of RBC, HGB and MCHC were prominently increased by GFNPs treatment. It is worth mentioning that the GFNPs could improve the level of HGB by 35.7% after 5 days treatment. Our results demonstrated that the GFNPs could improve the levels of RBC, HGB and MCHC more than those by EPO at the dosage used. In addition, the bone marrow pathology was used to precisely evaluate the anti-AA effects of GFNPs. The hematoxylin eosin (HE) staining exhibited that the extensive adipose (yellow arrow) was found in bone marrow of AA mice, compared with the abundant hematopoietic cells (red arrow) appearing in bone marrow of the control mice (Figure 2F). Surprisingly, the typically pathological changes in AA were obviously ameliorated by GFNPs, and there was a great increase of hematopoietic cells as well as a decrease of adipose. Thus, we suggest GFNPs have a superior effect on anti-AA.

### The effects of GFNPs on the differentiation and development of erythrocyte in bone marrow of AA mice

**Figure 3:**
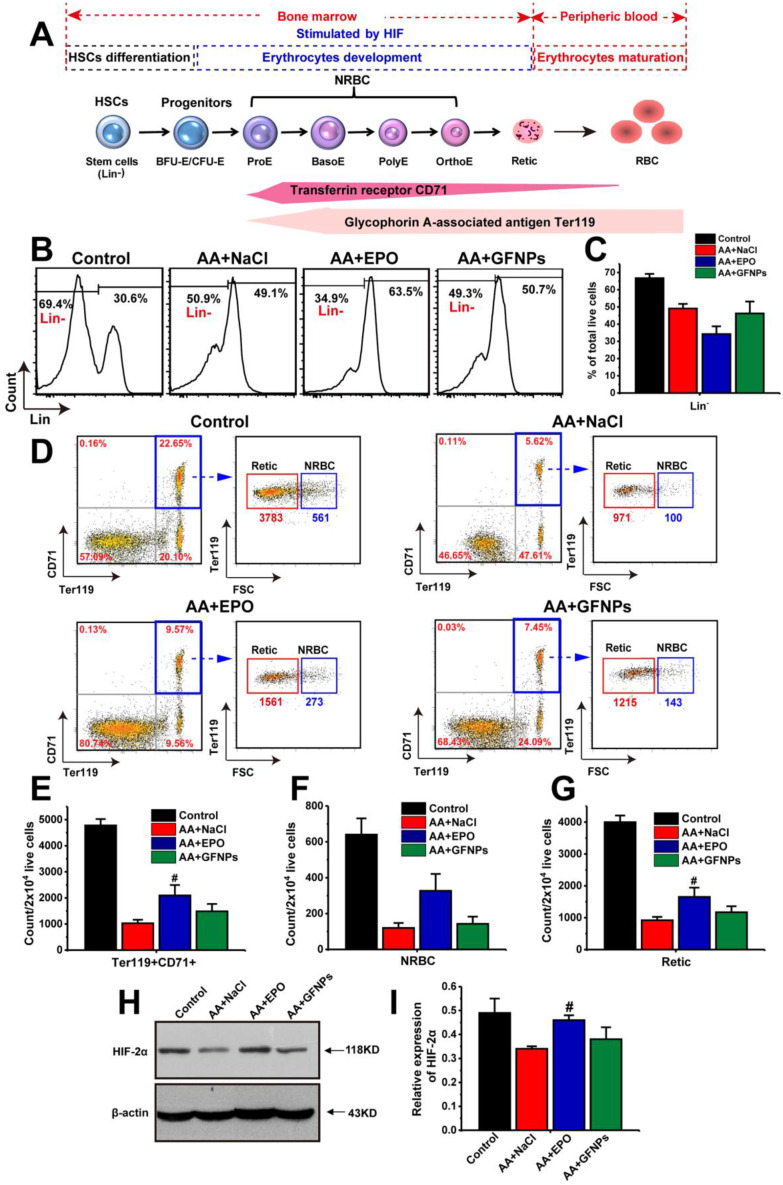
** The effects of GFNPs on the erythrocyte differentiation and development in bone marrow of AA mice. (A)** The schematic representation of the erythropoietic stages in bone marrow and peripheral blood. **(B)** The immature hematopoietic cells in bone marrow were specifically labeled by Lin^-^ marker. **(C)** The relative proportion of the Lin^-^ cells in total live cells. **(D)** The FCM analysis of Ter119^+^CD71^+^ cells (blue border), NRBC (Ter119^+^CD71^+^FSC^+^) and Retic (Ter119^+^CD71^+^FSC^-^) in bone marrow. **(E-G)** The counts of Ter119^+^CD71^+^ cells, NRBC, and Retic in 2x10^4^ live cells. **(H-I)** The protein expression of HIF-2α in bone marrow. (n = 5; ^#^ p < 0.05, ^##^ p < 0.01, vs AA+NaCl group).

Encouraged by the above results, we moved on to investigate the anti-AA mechanism by studying the effects of GFNPs on the whole process of erythropoiesis: hemopoietic stem cells (HSCs) differentiation, erythrocyte development and maturation 4,32,33. The first two stages occur in the bone marrow, and the process of erythrocyte maturation primarily takes place in the peripheral blood (Figure 3A). The HSCs differentiation is the initial stage of hematopoiesis and hematopoietic cells development. The population of immature hematopoietic cells during hemopoietic differentiation could be measured using FCM labeled by the lineage-negative (Lin-) specific maker 34,35. Our results demonstrated that the population of Lin- cells in AA mice was markedly decreased compared with the normal mice (Figure 3B and C). As one of the physiological functions of EPO was to promote the differentiation of erythroid stem cells into erythroblast 36, we found that the population of Lin- cells in the EPO treated AA mice was notably decreased. Though the significant improvement on RBCs by GFNPs treatment, they had little influence on the HSCs differentiation. Then, the HSCs give rise to erythroid progenitors and further into erythroid precursors (also called the nucleated red cells, NRBC) and reticulocyte (Retic), called erythrocyte development. CD71, as known as a transferrin receptor, is highly expressed in NRBC and absent in mature erythrocytes 37. TER119 is an erythroid-specific marker expressed at all stages of differentiation from early proerythroblasts to mature erythrocytes 38. The forward scattered (FSC) is commonly used to reflect relative cell size in the FCM measurement, and the size of Retic was smaller than that of NRBC. Thus, we could use CD71 and Ter119 two makers combining with FSC to quantitatively identify the proportion of NRBC (CD71^+^Ter119^+^FSC^+^) and Retic (CD71^+^Ter119^+^FSC^-^) by FCM. Our data revealed that due to the side-effects of chemotherapy drugs on erythropoietic stages, the total content of immature erythrocytes (CD71^+^Ter119^+^) include NRBC and Retic were sharply declined in AA mice (Figure 3D and E), and the respective counts of NRBC and Retic in 2×10^4^ live cells were also decreased (Figure 3F and G). After EPO treatment, the counts of CD71^+^Ter119^+^ cells, NRBC and Retic were all boosted owing to the stimulating hematopoietic functions of EPO. Contrastively, GFNPs had little impact on the counts of CD71^+^Ter119^+^ cells, NRBC and Retic in bone marrow of AA mice.

Additionally, the erythrocyte development was primarily adjusted by hypoxia-inducible factor-2α (HIF-2α). HIF-2α plays a vital role in hematopoiesis, and it regulates murine hematopoietic development in an erythropoietin-development manner. Our results revealed that the protein expression of HIF-2α was declined sharply in AA mice compared with the control (Figure 3H and I). It was increased in EPO-treated mice, but we found that GFNPs also did little to regulate the relative expression of HIF-2α, which further confirmed the above conclusion. Together, we considered that GFNPs could hardly contribute to the differentiation and development of erythrocyte in AA mice induced by chemotherapy, while EPO performed well on these processes. Thus, we didn't conduct further studies on the effect of GFNPs on these processes in bone marrow and inferred that the mechanism of GFNPs on AA could be different from that of EPO. We continued to study the other hematopoietic process-erythrocyte maturation in peripheral blood.

### The effect of GFNPs on the erythrocyte maturation in peripheral blood of AA mice

**Figure 4:**
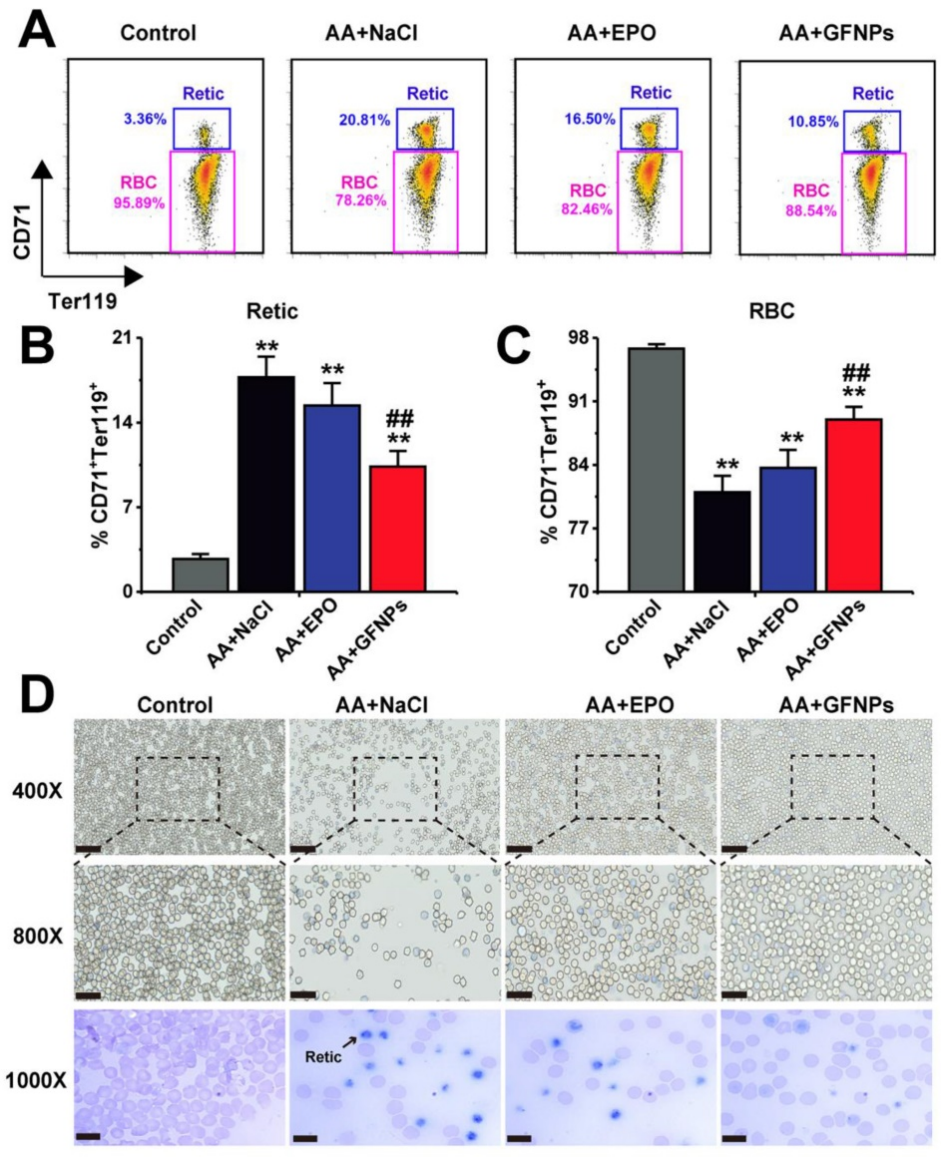
** GFNPs promoted the erythrocyte maturation of AA mice in blood. (A)** The FCM analysis of Retic (CD71^+^Ter119^+^) and RBC (CD71^-^Ter119^+^) in blood. The relative proportion of **(B)** Retic and **(C)** mature RBC in all groups. **(D)** The reticulocyte staining in blood. The Retic were dyed reticular blue. Scale bar = 50 µm (top), 25 µm (middle) and 10 µm (bottom) (n=5; ^*^ p < 0.05, ^**^ p < 0.01, vs Control group; ^#^ p < 0.05, ^##^ p < 0.01, vs AA + NaCl group).

The Retic are released from bone marrow into the peripheral blood developing into mature RBC, called as erythrocyte maturation. In normal mice, there was no more than 5% retic in blood. However, due to the dysfunction of erythrocyte development and maturation induced by chemotherapy drugs, the relative proportion of Retic (CD71^+^Ter119^+^) in blood of AA mice was significantly higher than that in the normal mice (20.81% vs 3.36%, Figure 4A and B). Excitedly, we found that GFNPs could greatly promote the erythrocyte maturation of peripheral blood in AA mice (Figure 4C), leading to more RBC (CD71^-^Ter119^+^) in blood. We also found that the improvement on erythrocyte maturation by EPO treatment was notably lower than that by GFNPs. The blood smear method was further performed to observe Retic in blood by new methylene blue (NMB) and eosin staining (Figure 4D). As Retic contain abundant basophilic RNA before mature, which could be dyed reticular blue by NMB 39,40. After denucleation and mature, RBC are only dyed by eosin instead of by NMB. It was clearly to reveal that the Retic were markedly reduced and the RBC was observably increased in blood by GFNPs treatment. But there were lots of Retic in AA mice treated both by normal saline and EPO, which was well consisted with the above results. All these data suggested that GFNPs highly promoted the erythrocyte maturation in the peripheral blood, superior to the effect of EPO. Driven by curiosity, we continued to conduct further studies in the peripheral blood.

**Figure 5:**
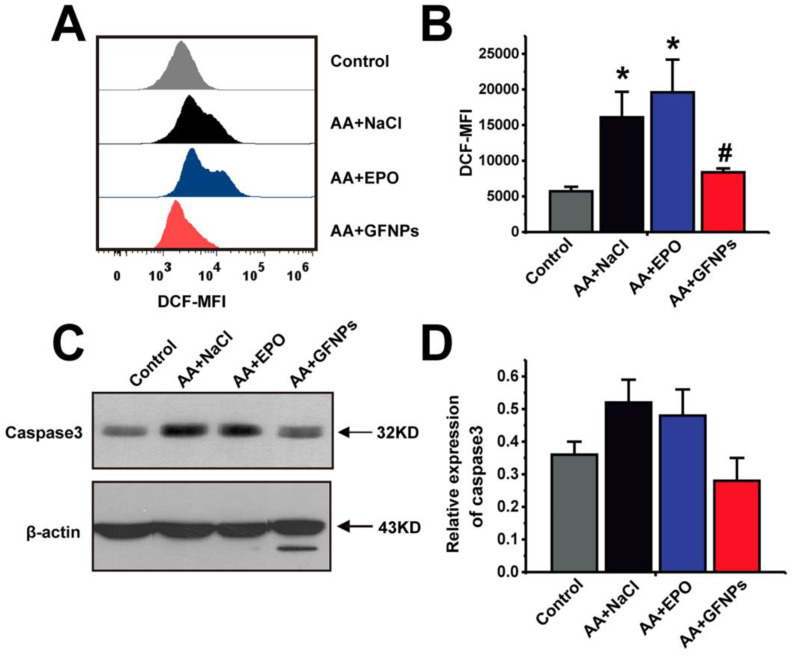
** GFNPs decreased the ROS levels and inhibited apoptosis of blood cells in peripheral blood of AA mice. (A)** The FCM analysis of the ROS levels in total blood cells of mice in Control, AA + NaCl, AA + EPO, and AA + GFNPs group respectively. **(B)** The total ROS levels in blood. **(C-D)** WB analysis of the Caspase 3 expression in whole blood. (n=5; ^*^ p < 0.05, vs Control group; ^#^ p < 0.05, vs AA + NaCl group).

Given that AA induced by chemotherapy drugs is closely associated with ROS and GFNPs have the excellent performance on scavenging the excess free radicals in the body, we continued to study the effect of GFNPs on the ROS level in the peripheral blood of AA mice. Once the steady state of peripheral blood was disturbed by chemotherapy drugs in AA mice, it generated excessive ROS of blood cells measured by FCM (Figure 5A and B). We found that the ROS levels of blood cells were notably reversed by GFNPs treatment. However, the EPO barely affected the level of ROS in blood. The excessive ROS in AA mice further induced the apoptosis of blood cells resulting in the high protein expression of Caspase 3 measured by western blotting (WB) (Figure 5C and D). And Caspase 3 is an effector of apoptosis and the final executor of apoptosis 41-43. Interestingly, GFNPs could remarkably decrease the protein expression of Caspase 3. The EPO also had little effects on Caspase 3. Mechanically, these results suggested that GFNPs decreased the excessive ROS and apoptosis of blood cells in AA mice.

### The biodistribution study and toxicity evaluation of GFNPs *in vivo*

**Figure 6:**
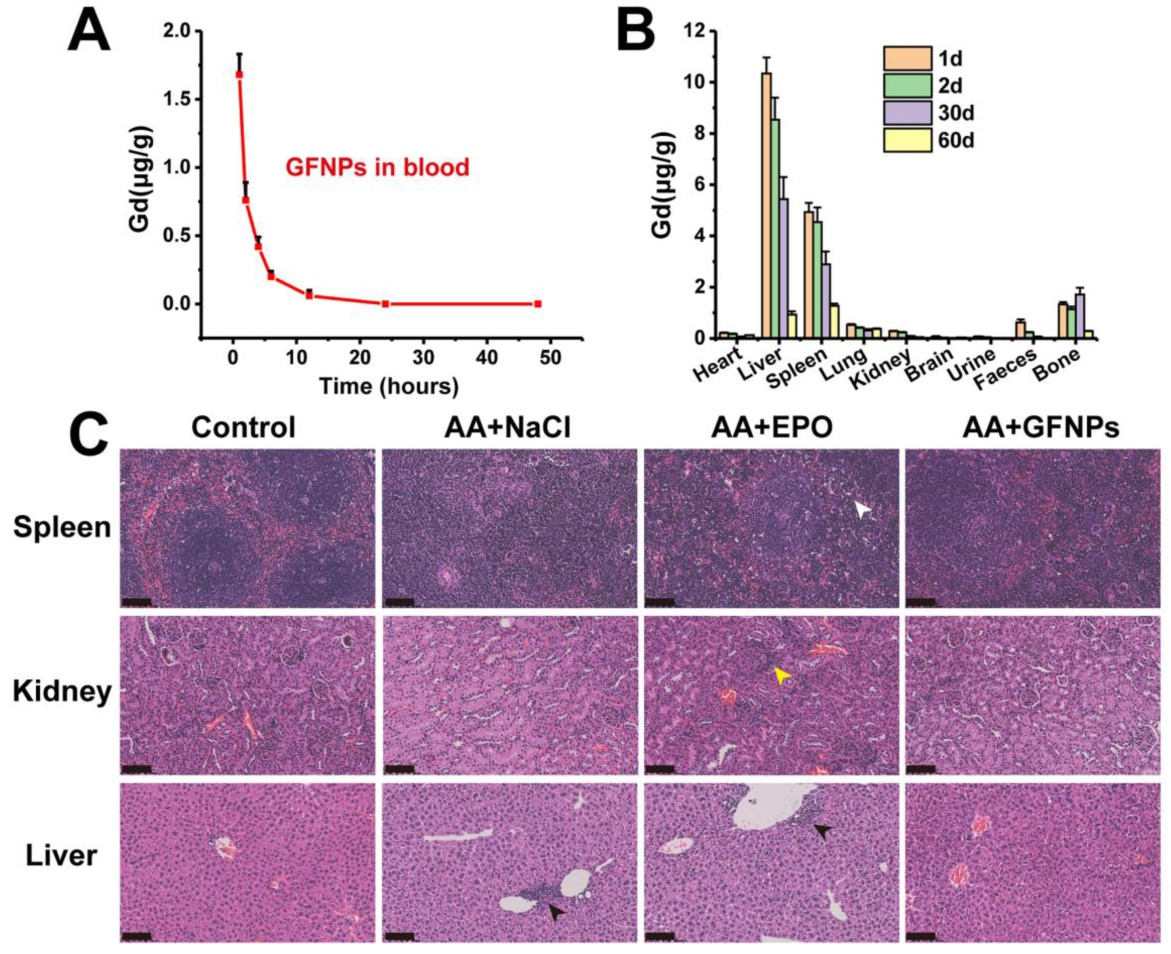
** (A)** The blood concentrations of GFNPs *in vivo*. **(B)** The distribution and metabolism of GFNPs *in vivo*. **(C)** The pathologic analysis of spleen, kidney, and liver of mice, Scale bar = 100 µm (n = 5).

The biodistribution study of GFNPs was performed by the inductively coupled plasma mass spectrometry (ICP-MS). It has showed that the concentration of Gd^3+^ declined gradually in blood after 24 h treatment of GFNPs, and the half-life of GFNPs in blood was ~2.95 h (Figure 6A). In addition, the GFNPs were accumulated into liver, spleen, and bone, which were mostly eliminated from the living body 60 days after administration (Figure 6B). Meanwhile, to evaluate the safety of GFNPs on anti-AA therapy, we carried on to investigate the histopathological examinations of main tissues and organs at the end of anti-AA treatment (the 15th day). The H&E pathologic results showed that there was no obvious toxicity of GFNPs towards the main organs (spleen, kidney, and liver) after anti-AA therapy (Figure 6C). However, there existed some abnormities in EPO-treated AA mice, which performed as inflammation & necrosis of spleen (white arrow), abnormal glomeruli of kidney (yellow arrow) and nuclear accumulation of hepatic cell (black arrow). Notably, to further evaluate the hepatic and nephrotic toxicity of GFNPs after anti-AA therapy, we conducted the blood analysis including alanine aminotransferase (ALT), aspartate aminotransferase (AST), alkaline phosphatase (ALP), uric acid (UA), and blood urea nitrogen (BUN) on day 15. As for the hepatic function, the level of ALT, AST, and ALP in AA group (Table S1) were abnormal compared with those in Control group, especially the level of AST, which decreased significantly in AA group. Meanwhile, the levels of UA and BUN for nephrotic function evaluation in AA group were decreased obviously compared with those in Control group. These suggested it induced certain toxicity towards the liver and kidney after the administration of chemotherapy drugs. Inversely, the relevant indices for hepatic and nephrotic function in AA+GFNPs group had prominently improved compared with those in AA group, which almost reversed back to the normal. Together, these results demonstrated GFNPs could be cleared from main organs as time advanced and decreased the toxicity during AA treatment.

## Conclusion

In summary, GFNPs were found to serve as a versatile nanoparticle with excellent performance of anti-oxidation and anti-AA therapeutic effect in mice. When GFNPs were intravenously injected into AA mice for six consecutive days, the counts of RBC in blood were significantly increased and the pathological state of bone marrow was notably ameliorated. Remarkably, though GFNPs had little impact on the differentiation of hematopoietic cells or the development of erythrocytes, it was amazing that GFNPs could promote the erythrocyte maturation in blood obviously. Further, GFNPs could strikingly decrease the ROS level and inhibit apoptosis of blood cells in peripheral blood of AA mice. Besides, GFNPs could be excreted from the mice, causing no serious toxicity. This work provided a new inspiration for the treatment of anemia as well as other blood diseases and further alleviating the side-effects of chemotherapy using the functionalized nanoparticles.

## Materials and methods

### Materials

Gd@C_82_ (99% purity) was purchased from Xiamen Funano Co. Ltd. (Xiamen, China). Dulbecco's minimal essential medium (DMEM), 2',7'-Dichlorodihydrofluorescein diacetate (DCFH-DA), and PBS were purchased from BioDee Biotechnology Co. Ltd. (Beijing, China). NaOH, HNO_3_, and H_2_O_2_ were purchased from Beijing Chemical Works (Beijing, China). 5, 5-dimethyl-1-pyrroline-N-oxide (DMPO), Busulfan (BUS) and Cyclophosphamide (CTX) were obtained from Sigma-Aldrich (MO., USA). Chemicals used were all analytical reagents and solvents without further purification.

### Preparation of gadofullerenes nanoparticles (GFNPs)

The water-soluble GFNPs were prepared as our previous studies 26,27. Simply, the suspension of 50 mg Gd@C_82_ in 30% of aqueous H_2_O_2_ (7 mL) and 12% NaOH (3 mL) were vigorously stirred under 50 ^o^C until the black suspension was dissolved gradually into a brownish-red solution. The solution was precipitated by ethanol and centrifuged at 9000 r/min for 5 min, and then the supernatant was discarded repeated for three times. Before used, the samples were further purified by dialysis (M.W.= 3500 Da) against ultrapure water (Millipore, Billerica, USA) to get rid of the unreacted materials.

### Characterization of GFNPs

Inductively coupled plasma-mass spectrometry (ICP-MS, Thermo, iCAP-Q, USA) was conducted to determine the concentration of GFNPs. Fourier transform infrared spectrum (FT-IR, Nicolet iN10 MX, Thermo, USA) and X-ray photoelectron spectroscopy (XPS, ESCALab220i-XL, VG, U.K.) were taken to characterize the chemical structure and average composition of GFNPs after freeze-drying. Besides, the morphology characterizations of GFNPs were conducted by atomic force microscope (AFM, Bruke, Multimode 8, Germany). The particle size and Zeta potential of GFNPs in water were characterized by dynamic light scattering (DLS, Malvern, Nano-ZS90, UK).

Electron spin-resonance (ESR, JES-FA200, JEOL, Japan) was conducted to examine the hydroxyl radical scavenging capacity of GFNPs *in vitro*. Simply, 40 μL H_2_O_2_ (100 mM) and 20 μL DMPO (100 mM) were mixed with 20 μL GFNPs (30 μM) or ultrapure water, respectively. DMPO acted as a trapping agent and H_2_O_2_ was used to produce hydroxyl radical. The mixture was irradiated by a lamp (UV, 500 W, 4 min), and then the data were collected in dark.

A mouse bone marrow cell (FDC-P1 cell) was obtained from National Infrastructure of Cell Line Resource (Shanghai, China). The cells were incubated with DMEM-H (Invitrogen, California, USA) which was added the 10% fetal bovine serum (FBS, Hyclone Co., South Logan, UT), 2×10^-3^ μM IL-3 (Gibco, NY, USA) and 1% penicillin & streptomycin.

The cytotoxicity assessment of GFNPs was conducted as the conventional method. Briefly, 100 μL 1×10^5^ mL^-1^ FDC-P1 cells were incubated in a 96-well transparent plate for 24 h, then added 100 μL GFNPs (0, 1, 2, 4, 10, 20, 40, 100 μM) for another 24 h. Afterwards, the cell viability of FDC-P1 cells were examined according to a Cell Counting Kit-8 (CCK-8, DOJINDO, Kumamoto, Japan).

The antioxidant capacity of GFNPs was tested as follows. 1 mL 1×10^5^ FDC-P1 cells were seeded in a 6-well transparent plate for 24 h, then added 1 mL pure culture medium or culture medium with H_2_O_2_ (400 μM) for 1 h. Subsequently, the medium was renewal with new medium with different concentrations of GFNPs (0, 20, 40, 100 μM) for 3 h. Afterwards, FDC-P1 cells were cultured with 500 μL 2',7'-Dichlorodihydrofluorescein diacetate (DCFH-DA, 10 μM, Beyotime, China) at 37 °C in dark for 30 min, and then washed three times. Mean fluorescence intensity (MFI) of DCF was measured to quantize the level of intracellular reactive oxygen species by a flow cytometer (Invitrogen, AttuneTM NxT, USA). The detection channel of flow cytometer is BL1, and the excitation & emission wavelength are 488 nm and 530/30 nm, respectively.

### Animal Experiments

Female ICR mice (24-26 g) were maintained from Sibeifu Beijing Biotechnology Co. Ltd. (Beijing, China). All the animal experiments were carried out in accordance with the National Regulation of China for Care and Use of Laboratory Animals, and approved by the Animal Ethics Committee of Institute of Processes, Chinese Academy of Sciences. Before the experiments, mice were acclimatized to laboratory conditions for 1 week. And after the states of mice were stable, the experiments were started as the day 0. All the animals were provided free access to food and water.

### Toxicity study *in vivo*

Mice in two groups were treated as follows (n=5): 1) GFNPs group was injected GFNPs solution (150 μL, 60 mM/kg/day) by intravenous injection (i.v.) for six continuous days on day 9-14; 2) NaCl group was injected 0.9% NaCl (150 μL) by i.v. on day 9-14. The hematological toxicity studies on day 0, 8, 12, 15 were carried out. Subsequently, on day 15, the heart, liver, spleen, lung and kidney were obtained from the mice. Afterwards, the hematoxylin and eosin staining for histological studies were conducted.

### The aplastic anemia (AA) therapy* in vivo*

20 female ICR mice were divided into four groups randomly (n = 5, each group). 1) Control group was i.v. 0.9% NaCl (150 μL) on day 9-14; 2) AA + NaCl group was administered BUS (20 mg/kg) and CTX (80 mg/kg) by intraperitoneal injection (i.p.) on day 1, 3, 5, 7, then i.v. 0.9% NaCl (150 μL) on day 9-14; 3) AA + EPO group was i.p. BUS and CTX on day 1, 3, 5, 7, then i.v. EPO (150 μL, 6×10^4^ IU/kg/day) on day 9, 11, 13; 4) AA + GFNPs was i.p. BUS and CTX on day 1, 3, 5, 7, then i.v. GFNPs (150 μL, 60 mM/kg/day) on day 9-14. The other operations were consistent with the Control group. On day 15, the mice were sacrificed by cervical dislocation to obtain blood and tissues for further studies.

### Hematological analysis

20 μL peripheral blood samples (the 15th day) were collected into anticoagulant tubes. Four important indicators including red blood cell (RBC), hemoglobin (HGB), and mean corpuscular hemoglobin concentration (MCHC) were examined using automated hematology analyzer (Mindray, BC-5000vet, China).

About 1 mL peripheral blood samples (the 15th day) were collected and centrifuged to obtain the serum for biochemical analysis. The serum levels of relevant indicators including alanine aminotransferase (ALT), aspartate aminotransferase (AST), alkaline phosphatase (ALP), uric acid (UA), and blood urea nitrogen (BUN) were detected to evaluate the hepatic and nephrotic toxicity of GFNPs using the automatic biochemical analyzer (Toshiba, TBA-2000FR, Japan).

### Histopathological Examination

The tissues (femur, heart, spleen, kidneys, liver, and lung) were harvested at the end of the animal experiments, and fixed in 4% formalin solution. Then the tissues were respectively dehydrated, embedded in paraffin, cut into 5-μm-thick slices and stained with hematoxylin and eosin (H&E). Subsequently, the slices were scanned using a scanning microtome (KF-PRO-005, KFBIO, China). The histopathological tests were performed according to a standard laboratory procedure. Each sample was for conventional processing and analysis.

### Flow cytometry

Bone marrow cells and peripheral blood cells were collected and re-suspended in PBS (1×) on day 15. For hematopoietic cells phenotypic analysis, 1×10^6^ bone marrow cells were incubated with hematopoietic lineage antibody (eBioscience, #88-7772-72) at 4 °C for 1 h in the dark. For the analysis of erythrocyte development and changes of ROS in related process, the BM cells were stained with Ter 119-PE (1:400, BioLegend, AB_313709) and CD71-Percp cy5.5 (1:400, BioLegend, AB_2565482) for 30 minutes in the dark, then incubated with 10 μM DCFH-DA for 30 min at 37 °C, subsequently, washed and re-suspended in PBS. With regard to erythrocyte maturation process in blood and changes of ROS in related process, 3 μL PB cells were diluted with 50 μL PBS (1×), then stained with Ter 119-PE (1:400) and CD71-Percp cy5.5 (1:400) for 30 minutes in the dark, incubated with DCFH-DA (10 μM) for 30 min at 37 °C, and washed, re-suspended in PBS (1×). Finally, cells were measured in the flow cytometer. The results were analyzed by using FlowJo 7.6 software. The detection channel of flow cytometer is BL1-BL4, and the excitation wavelength is 488 nm.

### Western Blotting

Western blotting (WB) was performed to quantify the protein expression of hypoxia-inducible factor-2α (HIF-2α, Abcam, AB199) in bone marrow, and Caspase 3 (GeneTex, GTX110543) in blood. β-actin (ZS, TA-09) was performed as an internal reference protein. Briefly, blood cells and bone marrow cells were collected and lysed in radio immunoprecipitation assay (RIPA) buffer (Beyotime, China), and then Bicinchoninic Acid (BCA) protein assay (Thermo, USA) was used to determine protein concentration. 40 μg protein extracts were separated via polyacrylamide gel electrophoresis (PAGE) electrophoresis, and then transferred onto polyvinylidene fluoride (PVDF) membranes (0.45 μm, Millipore, USA). The membranes were blocked with 5% non-fat milk for 1 h under agitation, and then incubated with the primary antibody overnight. Subsequently, after washing 3 times with Tris Buffered Saline Tween (TBST), the membranes were incubated with the secondary antibody for 40 minutes at room temperature. Finally, enhanced chemiluminescence (ECL) detection reagents (Millipore, USA) were used to develop the protein, and Gel Image system ver.4.00 (Tanon, China) was carried out for quantifying the gray level, and β-actin bands were used for normalization.

### Reticulocyte staining

To study the effect of GFNPs on the mature/immature erythrocytes in peripheral blood intuitively, blood cells were stained with reticulocyte staining solution (Baso Co. Ltd., ZUH, China). And then pictures were taken using a scanner (KF-PRO-005, KFBIO, China).

### Biodistribution study *in vivo*

The blood samples were collected at 1 h, 2 h, 4 h, 6 h, 12 h, 24 h, 48 h, and the mice were sacrificed on day 1, 2, 30, 60 after i.v. GFNPs (150 μL, 60 mM/kg). Then, the collected tissues (heart, liver, spleen, lung, kidney, brain, urine, faeces, and bone) and blood were weighed, digested by 1 mL HNO_3_ for at least 12 h till the tissues were dissolved. Subsequently, the samples were diluted with H_2_O 20~50 times, filtered with 220 nm membrane and detected by ICP-MS for the Gd^3+^ concentration.

### Statistical Analysis

Quantitative data reported were mean ± s.e.m, unless otherwise noted. Student's t-test was performed to assess statistical significance of the results and p < 0.05 was considered statistically significant.

## Supplementary Material

Supplementary figures and tables.

## Abbreviations

AA: aplastic anemia
HGB: hemoglobin
RBC: red blood cells
HSCs: hematopoietic stem cells
NRBC: nucleated red blood cells
Retic: reticulocytes
EPO: erythropoietin
HSCT: hematopoietic stem cell transplantation
IST: immunosuppressive therapy
ROS: reactive oxygen species
GFNPs: gadofullerene nanoparticles
WBC: white blood cells
AFM: atomic force microscope
DLS: dynamic light scattering
PDI: polydispersity index
FT-IR: fourier transform infrared spectrum
XPS: x-ray photoelectron spectroscopy
•OH: hydroxyl radical
ESR: electron spin-resonance
DMPO: 5-dimethyl-1-pyrroline-N-oxide
FCM: flow cytometry
HCT: hematocrit
i.p.: intraperitoneal
MCHC: mean corpuscular hemoglobin concentration
ALT: alanine aminotransferase
AST: aspartate aminotransferase
ALP: alkaline phosphatase
UA: uric acid
BUN: blood urea nitrogen
HE: hematoxylin eosin
Lin-: lineage-negative
FSC: forward scattered
HIF-2α: hypoxia-inducible factor-2α
NMB: new methylene blue
WB: western blotting
ICP-MS: inductively coupled plasma mass spectrometry
DMEM: dulbecco's minimal essential medium
DCFH-DA: 2',7'-Dichlorodihydrofluorescein diacetate
BUS: busulfan
CTX: cyclophosphamide
MFI: mean fluorescence intensity
RIPA: radio immunoprecipitation assay
BCA: bicinchoninic acid
PAGE: polyacrylamide gel electrophoresis
PVDF: polyvinylidene fluoride
TBST: tris buffered saline tween
ECL: enhanced chemiluminescence
