# Supplementary material for: Gadofullerene nanoparticles for robust treatment of aplastic anemia induced by chemotherapy drugs
Source: Theranostics. 2020 May 23;10(15):6886–97. doi: 10.7150/thno.46794 (PMC7295067; doi:10.7150/thno.46794)
Supplement: Supplementary file 1 — Supplementary figures and tables. [file thnov10p6886s1.pdf]

Supporting information

Gadofullerene nanoparticles for robust treatment of aplastic anemia induced by chemotherapy

Wang Jia [a,b], Mingming Zhen [a,b,*], Lei Li [a,b], Chen Zhou [a,b], Zihao Sun [a,b], Shuai Liu [a,b], Zhongpu Zhao [a,b], Jie Li [a,b], Chunru Wang [a,b,*] and Chunli Bai [a,b,*]

a. Beijing National Laboratory for Molecular Sciences, Key Laboratory of Molecular Nanostructure and Nanotechnology, CAS Research/Education Center for Excellence in Molecular Sciences, Institute of Chemistry, Chinese Academy of Sciences, Beijing 100190, China.
b. University of Chinese Academy of Sciences, Beijing 100049, China.

Corresponding Email: zhenmm@iccas.ac.cn, crwang@iccas.ac.cn, clbai@cas.cn

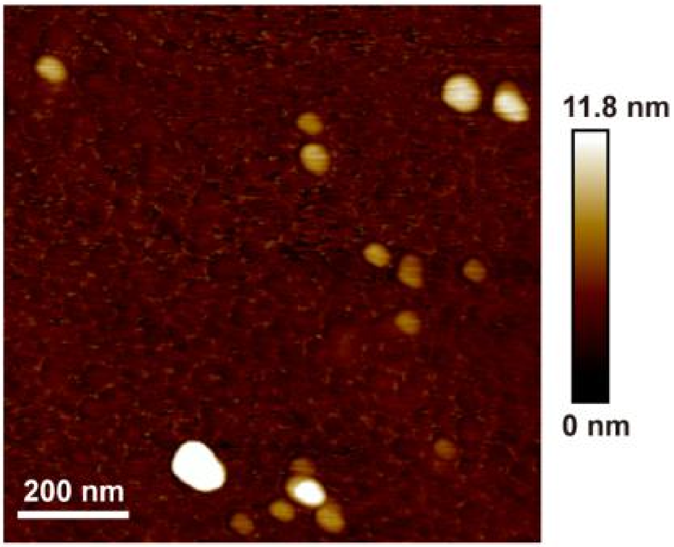

Figure S1. The AFM of GFNPs.

A

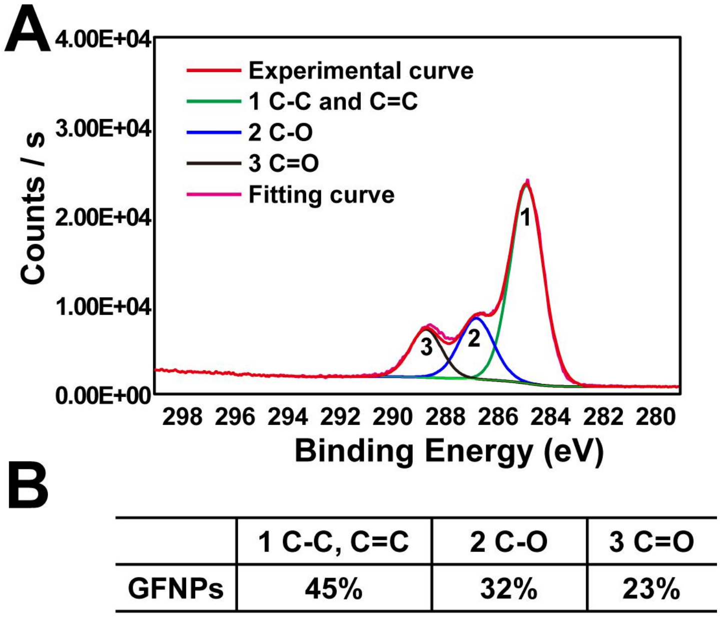

B

	1 C-C, C=C	2 C-O	3 C=O
GFNPs	45%	32%	23%

Figure S2. The XPS data of GFNPs. (A) The XPS spectrum of GFNPs. **(B)** The relative atomic percent contents of C1s (1 C-C, C=C; 2 C-O; 3 C=O) of GFNPs.

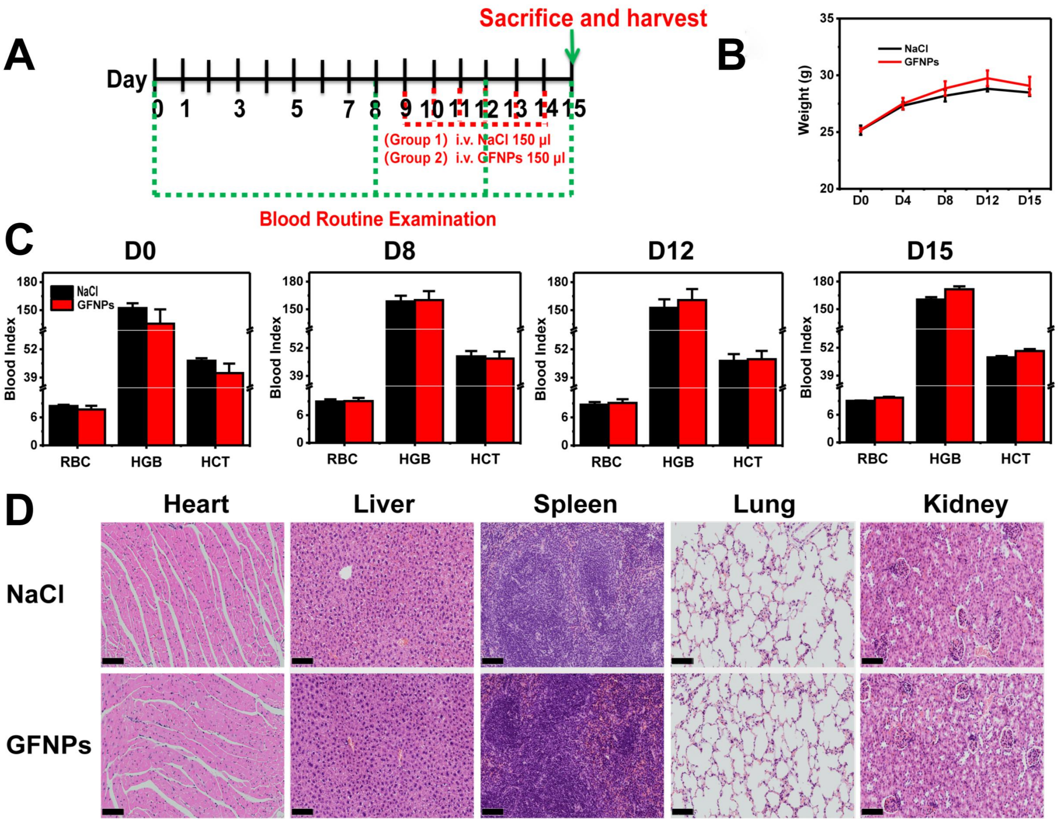

Figure S3. The safety evaluations of GFNPs *in vivo*. (A) Schedule of animal experiment treated with GFNPs/NaCl. **(B)** The changes of body weight during 15 days after i.v. injection GFNPs/NaCl. **(C)** The changes of hematological indices of mice in different times. **(D)** The light Microscopy of H&E sections of heart, liver, spleen, lung, and kidney. Scale bars are 100 μm.

Table S1. The blood analysis of hepatic and nephrotic toxicity of GFNPs. (n=5; * $p < 0.05$, ** $p < 0.01$, vs Control group)

Parameters	Groups		
	Control	AA	AA+GFNPs
ALT（U/L）	40.00±9.90	33.33±2.08	36.50±6.36
AST（U/L）	182.00±19.80	133.50±2.12*	199.00±9.92
ALP（U/L）	83.70±1.13	104.70±8.06*	92.75±8.27
UA（umol/L）	183.61±3.50	78.02±3.70**	122.39±29.31
BUN（mmol/L)	9.92±1.16	5.91±0.69*	6.98±0.64
